# Non-bacterial cystitis caused by pembrolizumab therapy for adenocarcinoma of the lung: a case report

**DOI:** 10.3389/fimmu.2024.1423123

**Published:** 2024-07-05

**Authors:** Caixia Di, Teng Yu, Lei Ni

**Affiliations:** ^1^ Department of Pulmonary and Critical Care Medicine, Shanghai Key Discipline for Respiratory Diseases, Institute of Respiratory Diseases, Ruijin Hospital, Shanghai Jiaotong University School of Medicine, Shanghai, China; ^2^ Key Laboratory of Emergency Prevention, Diagnosis and Treatment of Respiratory Infectious Diseases, Shanghai, China; ^3^ Department of Pathology, Ruijin Hospital, Shanghai Jiaotong University School of Medicine, Shanghai, China

**Keywords:** immunotherapy, lung cancer, pembrolizumab, cystitis, immune checkpoint inhibitors

## Abstract

Immune checkpoint inhibitors (ICIs) including anti-programmed death cell protein 1 (anti-PD1) and anti-programmed cell death-ligand 1 (PD-L1), by disinhibiting the antitumor responses of lymphocytes, have extended survival benefits for patients in lung cancer. ICIs can also lead to a wide spectrum of immune-related adverse events (irAEs), due to dysregulation of immune reactions. Here, we report a 27-year-old female patient with adenocarcinoma of the lung treated with pembrolizumab-combined chemotherapy treatment, who complained of urinary irritation symptoms. No bacteria were found in multiple urine cultures. B-mode ultrasonography indicated a high echo in the right lateral wall of the bladder, about 5.6 × 4.5 mm in size. Transurethral bladder tumor resection (TURBT) was operated. At biopsy, we found CD3^+^ CD8^+^ lymphocyte, plasma cell, and eosinophil infiltration and lymphoid follicle formation in the bladder mucosal layer. This is a report of non-bacterial inflammation of the urinary tract caused by immunotherapy.

## Introduction

The prognosis for patients with certain cancers has been significantly improved through the use of immune checkpoint inhibitors (ICIs). These monoclonal antibodies target specific immune checkpoints, such as cytotoxic T-lymphocyte-associated protein 4 (CTLA-4) and programmed cell death protein 1 (PD-1), thereby potentiating immune responses against cancer cells. Nevertheless, ICIs can also trigger immune-related adverse events (irAEs) across various tissues, a consequence of their mechanism of action. These irAEs encompass a range of conditions, including dermatological, gastrointestinal, endocrine, pulmonary, renal, ocular, rheumatological, and neuromuscular disorders. The majority of irAEs are acute and typically responsive to glucocorticoid therapy; however, some have been known to be fatal (1, 2). Non-bacterial cystitis represents a rare irAE, with the first case associated with pembrolizumab documented in 2020 (3). Currently, there is an unmet need for biomarkers that can facilitate the early detection of ICI-induced cystitis. We present here a case of non-bacterial cystitis occurring.

## Case presentation

**Figure 1:**
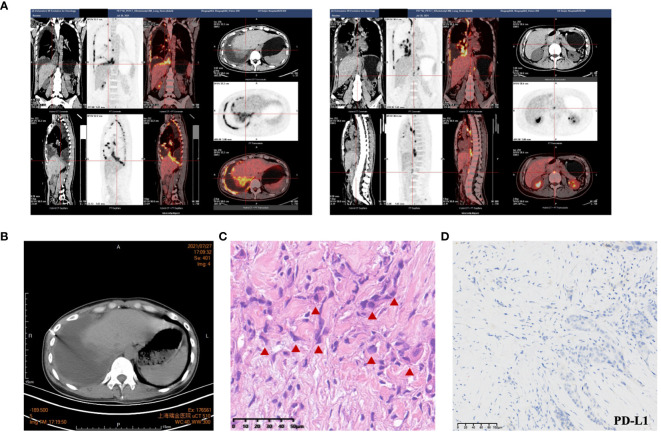
The iconography and pathology of lung adenocarcinoma. **(A)** PET-CT evaluation suggested the possibility of multiple metastases throughout the body (multiple lymph nodes throughout the body, posterior segment of the right lobe of liver, bone, and pleura). **(B)** CT-guided pleural biopsy. **(C)** HE staining indicated lung adenocarcinoma. **(D)** Immunohistochemistry (IHC) staining of the PD-L1 (22C3) of the lung adenocarcinoma. Significant abnormal findings were noted (red triangle).

**Figure 2:**
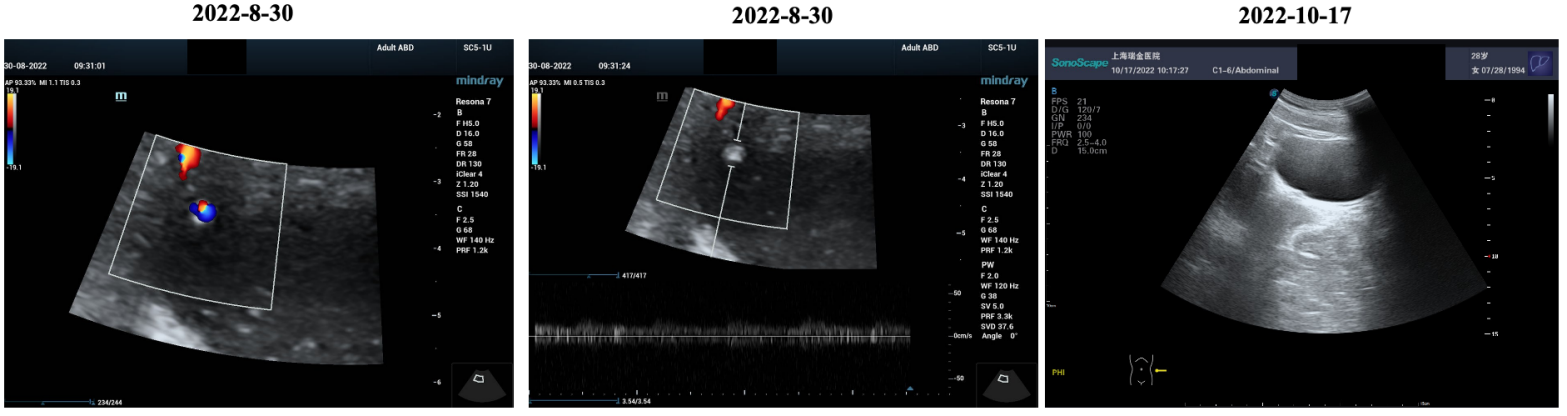
B-mode ultrasonography indicated a high echo in the right lateral wall of the bladder.

**Figure 3:**
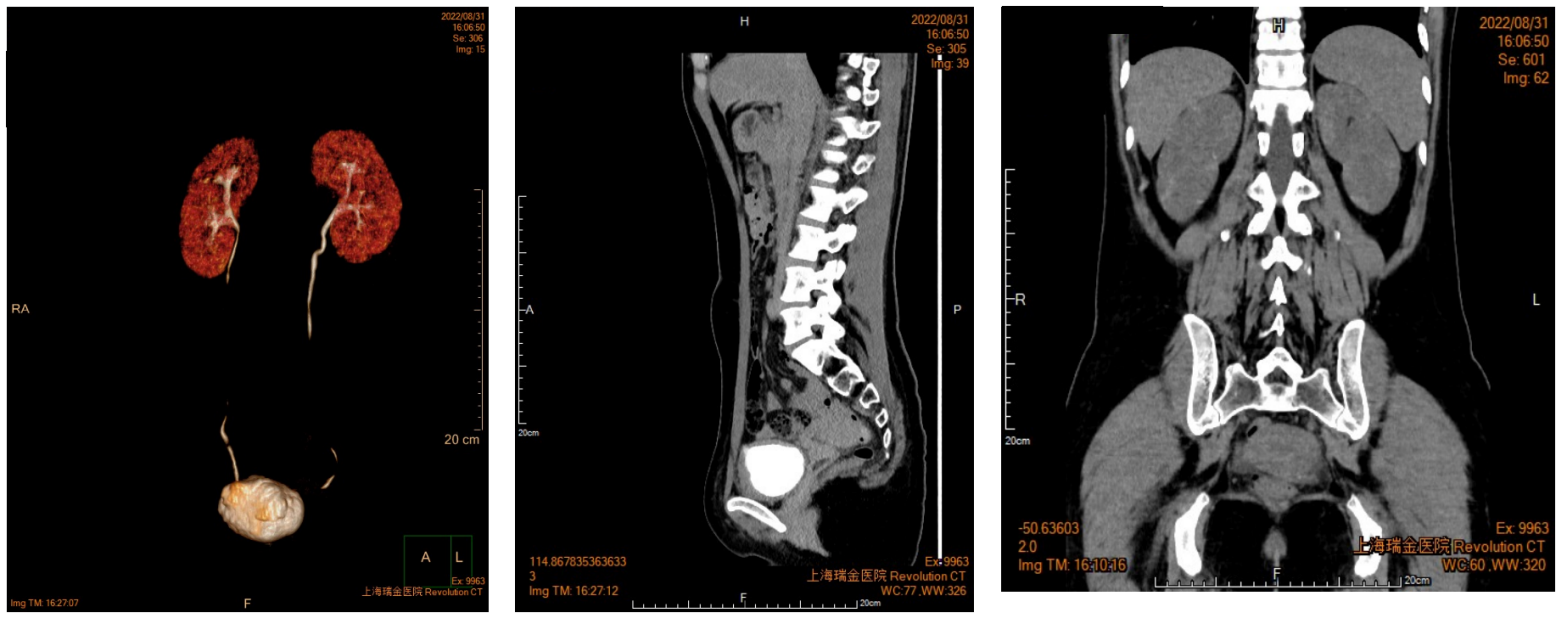
Enhanced CT of urinary system indicated multiple strips of slightly high density in both kidneys.

**Figure 4:**
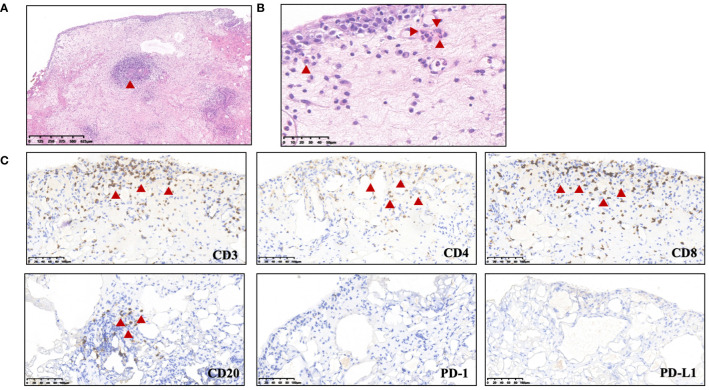
Biopsy of bladder occupation. **(A)** HE staining indicated depicting edematous and expanded lamina propria containing a mixed inflammatory infiltration (predominantly lymphocytes, plasma cells, and eosinophils) and focal lymphoid follicle formation. **(B)** Eosinophil infiltration. **(C)** Immunohistochemistry staining indicated lymphocyte infiltration.B lymphocytes, positive staining for CD20; T lymphocytes, positive staining for CD3. Significant abnormal findings were noted (Red triangle).

A 27-year-old woman visited a local hospital with a chief complaint of cough, exertional dyspnea after activity, and right chest pain in June 2021. Local chest computed tomography (CT) scanning showed a right pleural effusion. She received antibiotic therapy, but no improvement was observed. She was taken to our hospital for further diagnosis and treatment. Pleural effusion B-ultrasound and chest CT indicated encapsulated pleural hydrothorax, and after excluding contraindications, pleural hydrothorax puncture and catheter drainage were performed. A total of 2,575 ml yellow pleural hydrothorax was drained after surgery, and the exfoliated cells suggested the possibility of adenocarcinoma. Positron emission tomography/computed tomography (PET/CT) evaluation suggested the possibility of multiple metastases throughout the body (multiple lymph nodes throughout the body, posterior segment of the right lobe of liver, bone, and pleura) (**Figure 1A**). Exfoliated cell embedding pathology was performed, and CT-guided pleural mass puncture was performed on 03/08/2021 (**Figure 1B**). These findings led to a diagnosis of driver gene-negative adenocarcinoma of the lung (cT_4_N_3_M_1c_, stage IVB) according to the eighth edition AJCC staging system (**Figure 1C**). Immunohistochemistry (IHC) staining of the PD-L1 (22C3) showed that both the tumor proportion score (TPS) and combined positive score (CPS) were 0% (**Figure 1D**). Her Performance Status (PS) score was 1. Initially, the patient received six courses (08/2021 to 01/2022) of chemotherapy (pemetrexed and carboplatin) combined with pembrolizumab immunotherapy. Then, she was given maintenance therapy with pemetrexed combined with pembrolizumab for 2 cycles and subsequently pembrolizumab therapy for 2 cycles due to the COVID-19 pandemic. During first-line treatment, the best effect was assessed as partial response (PR), and the progression-free survival (PFS) was approximately 8 months (**Supplementary Figure 1**). Following the third and fourth cycle treatments, acute liver insufficiency appeared and was improved by using a hepatoprotective drug. After the fourth cycle, the menstrual disordered, onset of menstruation 15 days earlier. Chest CT on 07/2022 revealed that the tumor in the right lung showed progressive disease (PD) (**Supplementary Figure 2**). Meanwhile, MRI of the brain showed new metastasis in the brain (**Supplementary Figure 3**). The patient was given a second-line treatment of pemetrexed, pembrolizumab, and bevacizumab combined radiotherapy for left frontal metastases (35G/5Fx). Urinalysis results were normal before the first-line treatment. Urinary tract irritation including frequency of urination, hematuria, and painful micturition appeared after the first course of second-line therapy, whereas no fever was observed. She received cefoperazone sodium and sulbactam sodium injection but had no improvement. Urine examination revealed 1,630.6/μL white blood cells (WBCs) in the urine. Serum creatinine and urea levels remained normal. Urine culture indicated that no bacterial, fungal, or mycobacterium tuberculosis grew. Urinary exfoliation cytology suggests no high-grade urothelial carcinoma. Uronuclear matrix protein 22 test was negative. B-mode ultrasonography indicated that the bladder wall was rough, and the bladder was substantia occupying. There was a high echo in the right lateral wall of the bladder, approximately 5.6 × 4.5 mm in size, protruding into the bladder cavity with a papillary shape (**Figure 2**). Enhanced CT of the urinary system indicated multiple strips of slightly high density in both kidneys on plain CT scan, possible inflammatory changes, and thickening of bladder wall (**Figure 3**). Cystoscopy in other hospital indicated diffuse congestion with edema and thickening of the posterior wall and top of the bladder, and adenocarcinoma could not be ruled out. On 20/09/2022, transurethral bladder tumor resection (TURBT) was operated under general anesthesia. Microscopic examination revealed intact bladder mucosal tissue without atypia of the urothelium, with lymphocytic, plasmacytic, and eosinophilic infiltration, and focal lymphoid follicle formation in the stroma. The inflammatory infiltration stimulated capillary proliferation and edema of the bladder mucosal tissue. Immunohistochemistry staining supported a urothelial lineage including GATA-3, high-molecular-weight cytokeratins and CK20 of the urothelium, and mixed distribution of the B lymphocytes (positive staining for CD20, CD79a) and T lymphocytes (positive staining for CD3, CD5). It is worth noting that among the T lymphocytes, the amount of T lymphocytes positively staining for CD8 was much more than those positively staining for CD4, suggesting an abnormal immunological functioning status. The scattered plasma cells were outlined by CD38, further detecting the ratio of kappa and lambda showing no light chain limitation, and the ratio of IgG4 to IgG excluded the diagnosis of IgG4-related sclerotic lesions. All the above pathological evidence supported the diagnosis of chronic cystitis, reflecting the abnormal immunological functioning status (**Figure 4**). After the TURBT, the bladder irritation sign improved in a short time. However, the patient showed signs of recurrence of bladder irritation after pembrolizumab therapy again. After the multidisciplinary consultation, immune-related cystitis was suspected. Due to the age of the patient, the use of pembrolizumab therapy was continued. Interestingly, the bladder irritation sign disappeared when alectinib, as the third-line treatment, was given instead of pembrolizumab therapy and chemotherapy due to the ALK gene EML4~ALK fusion, which was found in the biopsy of lung by next-generation sequencing in response to progressive disease.

## Discussion

Here, we report a case of non-bacterial cystitis, a rare kind of irAE, caused by anti-PD-1 antibody at a patient with lung adenocarcinoma. We performed a comprehensive review of immune-related cystitis based on available case reports. For the cases reviewed, the demographic features, tumor types, clinical manifestations, and treatment were summarized (**Supplementary Table 1**). As shown in **Supplementary Table 1**, there are currently few reports of non-bacterial cystitis caused by ICIs for cancer treatment, including anti-PD-1 and anti-PD-L1 in patients with lung cancer (8/10), intrahepatic cholangiocarcinoma (ICC) (1/10), and advanced breast cancer (1/10). In addition, Toby Mur et al. reported a case of non-infective cystitis secondary to benralizumab treatment, a kind of antibody for IL-5 receptor α, for eosinophilic asthma and esophagitis (4). The defined timeframe for the development of non-bacterial cystitis is not clear now, which can range from 6 weeks to few years after the ICI treatment. The symptoms of immune-related cystitis include frequency of urination, hematuria, and painful micturition, few with pollakiuria and fever, which was similar to urinary tract infection caused by infection. It was the lack of specificity of symptoms that led to the difficulty to the diagnosis and differential diagnosis on the onset of the irAE. To date, there is no standardized diagnostic approach for immune-related cystitis.

The diagnosis relies on a biopsy to rule out other causes including bacterial infection, radiation-related cystitis, and metastasis. Histologic analysis usually indicated the bladder lymphocyte-dominant inflammation and interstitial tissue hyperplasia (5). Multidisciplinary discussion (MDT) by oncologist, urologist, radiologist, and pathologist is recommended to diagnosis. There were few reasons to diagnosis of immune-related cystitis in our patient. Firstly, she did receive radiation treatment for the head but not for the urinary system and not have etiological evidence. Secondly, she developed frequency of urination, hematuria, and painful micturition after an 11-month treatment of pembrolizumab. In addition, B-mode ultrasonography indicated chronic inflammation but a high echo which need to exclude metastasis. Therefore, a transurethral resection of bladder tumor (TURBT) was performed, and the biopsy showed chronic inflammation characterized by infiltration of multiple lymphocytes, plasma cells, and eosinophilic granulocytes, with the formation of focal lymphoid follicles, consistent with the pathology of cystitis. The signs of bladder irritation improved shortly after TURBT; however, they recurred following subsequent pembrolizumab therapy. After the multidisciplinary consultation, immune-related cystitis was considered.

Enhanced T-cell activity against shared antigens across normal and cancer cells is supported as a mechanism for irAE development by several preclinical models (6). The exact pathophysiological mechanism of immune-related cystitis is still unclear. Studies have shown that a history of autoimmune diseases may be associated with increased risk of irAEs (2, 7). Alessandro Ceschi et al. have reported that immunotherapy provokes the attack on antigens present on host non-cancer cells, which was termed as “on-target autoimmune toxicity” and “cytokine release syndrome”, which may be associated with immune-related cystitis (8). A previous case of pembrolizumab-induced cystitis raised the possibility that the unknown antigen in the urothelium is targeted by TIA^+^ and/or CD8^+^ lymphocytes (3). Toby Murray et al. reported that upregulation of cytokine pathways following the downregulation of IL-5 and subsequent eosinopenia may contribute to benralizumab induced immune-related cystitis (4). In our patient, we observed an increase of CD8^+^ CD28^+^ T cells in peripheral blood during the onset of cystitis (22.4%–24%, normal range 8.6%–15.5%), which may contribute to cystitis. A study by Lechner et al. revealed that clonally expanded, thyrotoxic effector CD8^+^ T cells driven by IL-21 contribute to checkpoint inhibitor thyroiditis (9). It was demonstrated that there was a prominent Th1-CD8^+^ T cell axis in both blood and inflamed joints of arthritis-irAEs (10). Recently, an early expansion of Ki-67^+^ regulatory T cells and Ki-67^+^ CD8^+^ T cells was found to likely be associated with an increased risk of irAEs in melanoma and non-small cell lung cancer (11). Interestingly, a study found that highly circulating CD8^+^ CD28^+^ T cell level is a potential biomarker for immunotherapy response and better prognosis, whereas excessive CD8^+^ CD28^+^ T cells may also indicate the emergence of severe irAEs, which may explain our findings in this study (12). Further detailed research, including both basic and clinical studies, is needed to elucidate the underlying pathophysiological mechanisms of immune-related cystitis.

Currently, there are no established common criteria for grading immune-related cystitis. For treatment of other irAEs, such as pneumonitis and myocarditis, of Grade 2 or higher, temporary immunosuppressive agents were needed according to treatment of irAEs in accordance with the Common Terminology Criteria for Adverse Events (CTCAE) (2). For immune-related cystitis, most patients were steroid-sensitive. However, Fukunaga et al. reported a steroid-resistant cystitis during treatment with nivolumab for lung cancer recently (13). Some patients may require discontinuation of ICIs. The optimal treatment, including the dosage and duration of steroid administration, remains a subject of debate.

Our report has several limitations. First, the patient did not undergo steroid treatment, which precludes us from evaluating whether the cystitis would have responded to steroid therapy. Secondly, since the cystoscopy was performed at another hospital, we are unable to provide the cystoscopy imaging data.

With the widespread use of ICIs in oncology, irAEs have raised lots of concerns in clinical practice. Clinicians should be able to evaluate and treat the heterogeneous manifestations of irAEs. Several studies have cataloged organ-specific biomarkers and non-organ-specific biomarkers of irAEs, including genotype, preexisting autoimmune disease, baseline autoantibodies, immune cell changes, and microbiome (14, 15). Importantly, some studies have identified a link between human leukocyte antigen (HLA) alleles and skin toxicity (16–18). In the context of pulmonary toxicity, high PD-L1 expression and increased counts of eosinophil and monocyte are risk factors in non-small cell lung cancer patients receiving ICI treatment (19). Early recognition of immune-related cystitis necessitates the development of potential organ-specific biomarkers. Further research involving larger patient cohorts with various cancer types, as well as studies at the animal and cellular levels, is imperative to exploring these putative biomarkers.

## Conclusion

In this report, we present a case of non-bacterial cystitis associated with pembrolizumab and provide a review of immune-related cystitis based on published case reports. Given the absence of specific symptoms and a standardized diagnostic criterion for immune-related cystitis, it is imperative for clinicians to be vigilant for its onset. Early recognition is essential to facilitate comprehensive assessment and multidisciplinary team management.

## Data availability statement

The original contributions presented in the study are included in the article/**Supplementary Material**. Further inquiries can be directed to the corresponding author.

## Ethics statement

The studies involving humans were approved by Ruijin Hospital Ethics Committee Shanghai Jiaotong University of Medicine. The studies were conducted in accordance with the local legislation and institutional requirements. The human samples used in this study were acquired from primarily isolated as part of your previous study for which ethical approval was obtained. Written informed consent for participation was required from the participants or the participants’ legal guardians/next of kin in accordance with the national legislation and institutional requirements. Written informed consent was obtained from the individual(s) for the publication of any potentially identifiable images or data included in this article. Written informed consent was obtained from the participant/patient(s) for the publication of this case report.

## Author contributions

LN: Conceptualization, Data curation, Investigation, Resources, Validation, Visualization, Writing – review & editing, Formal analysis, Project administration. CD: Conceptualization, Data curation, Formal analysis, Funding acquisition, Investigation, Methodology, Project administration, Validation, Writing – original draft. TY: Investigation, Methodology, Project administration, Writing – original draft.

## Supplementary material

Supplementary Figure 1 Clinical course of the patient.

Supplementary Figure 2 Chest CT revealed the dynamic change of the tumor in the right lung.

Supplementary Figure 3 Magnetic resonance imaging reveals left frontal lobe metastasis.

Supplementary Table 1 A detailed review of immune-related cystitis based on available case reports. NSCLC, non-small cell lung cancer; SCLC, small cell lung cancer; ICC, intrahepatic cholangiocarcinoma.
