# Supplementary figures and images for: Non-bacterial cystitis caused by pembrolizumab therapy for adenocarcinoma of the lung: a case report
Source: Front Immunol. 2024 Jul 5;15:1423123. doi: 10.3389/fimmu.2024.1423123 (PMC11257856; doi:10.3389/fimmu.2024.1423123)

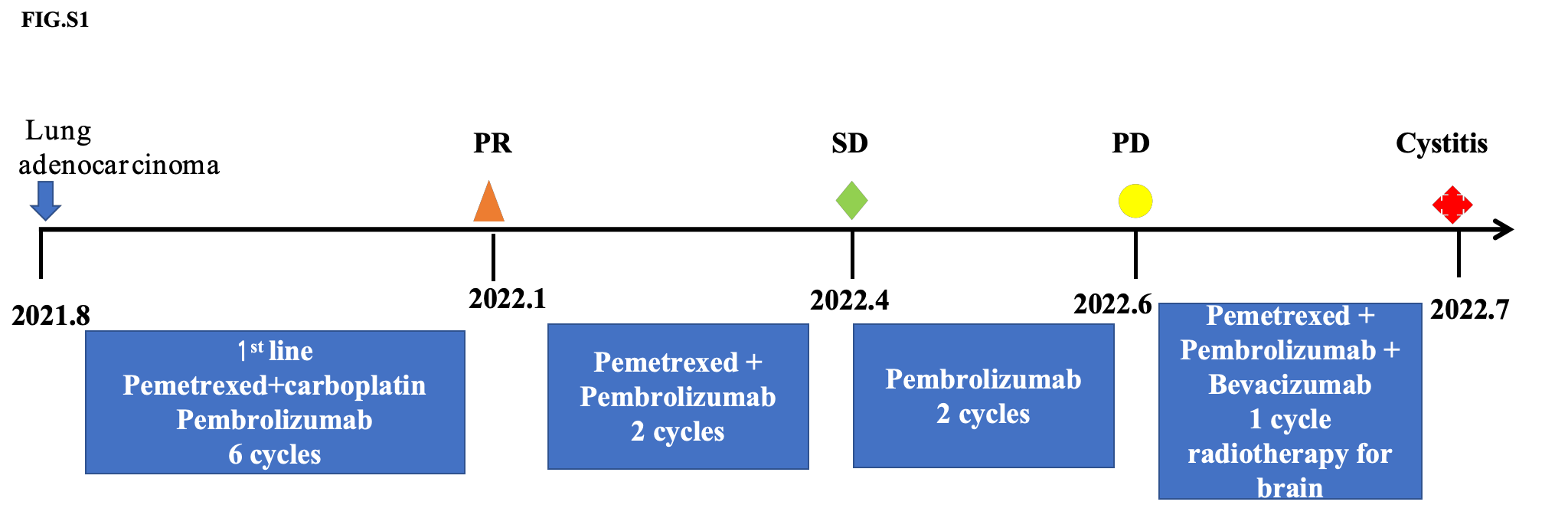

Supplement: Supplementary Figure 1 — Clinical course of the patient. [file Image_1.tif]

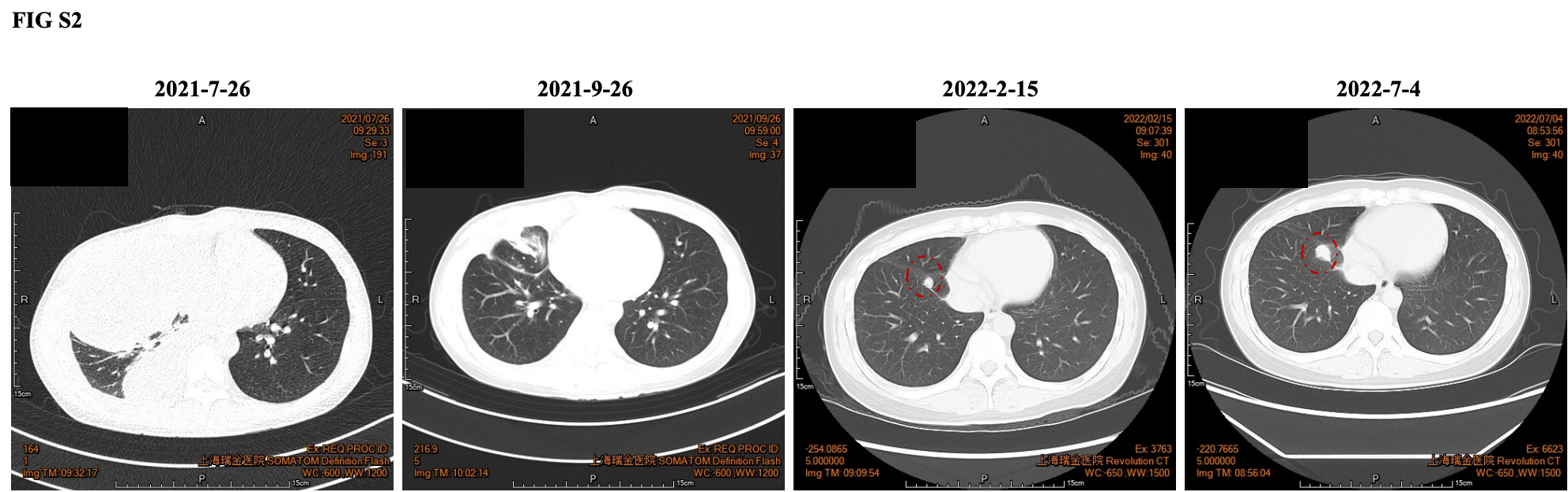

Supplement: Supplementary Figure 2 — Chest CT revealed the dynamic change of the tumor in the right lung. [file Image_2.tif]

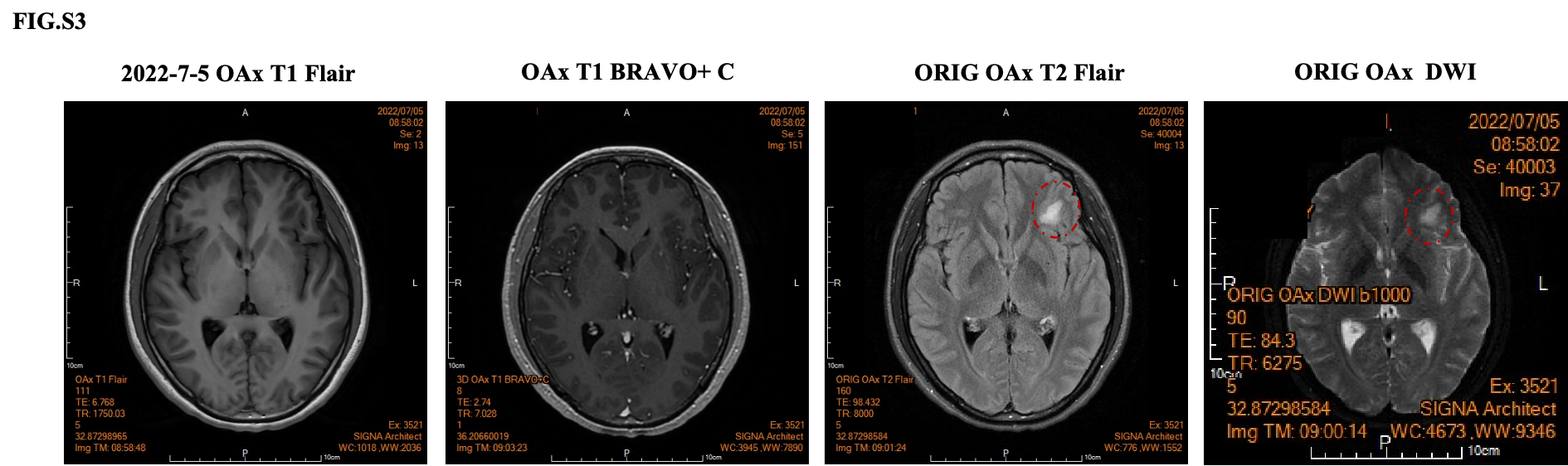

Supplement: Supplementary Figure 3 — Magnetic resonance imaging reveals left frontal lobe metastasis. [file Image_3.tif]
