# Supplementary material for: Non-bacterial cystitis caused by pembrolizumab therapy for adenocarcinoma of the lung: a case report
Source: Front Immunol. 2024 Jul 5;15:1423123. doi: 10.3389/fimmu.2024.1423123 (PMC11257856; doi:10.3389/fimmu.2024.1423123)
Supplement: Supplementary Table 1 — A detailed review of immune-related cystitis based on available case reports. NSCLC, non-small cell lung cancer; SCLC, small cell lung cancer; ICC, intrahepatic cholangiocarcinoma. [file Table_1.docx]

**Table S1** A detailed review of immune-related cystitis based on available case reports

| **Patient No** | **Age/sex** | **Malignancies** | **ICIs** | **Clinical presentations** | **Onset from ICIs** | **Treatment** | **Ref.** |
| --- | --- | --- | --- | --- | --- | --- | --- |
| **1** | 27/F | NSCLC | Pembrolizumab | Frequency of urination, haematuria, and painful micturition | 11 months | TURBT | Current paper |
| **2** | 51/M | SCLC | Nivolumab | Frequency of urination | 15 weeks | Methylprednisolone | Liping Zhu et al[1] |
| **3** | 78/F | NSCLC | Pembrolizumab | Pollakiuria and nocturia accompanied by painful micturition | 51 weeks | Prednisolone | Ueki Y et al[2] |
| **4** | 50/M | NSCLC | Nivolumab | Sudden onset pollakisuria, micturition pain, and diarrhea | 14 weeks | Prednisolone | Shimatani et al[3] |
| **5** | 60/M | NSCLC | Nivolumab | Pollakisuria, dysuria, and diarrhea | 24 weeks | Discontinuing nivolumab treatment | Shimatani et al[3] |
| **6** | 62/M | NSCLC | Nivolumab | Fever and diarrhea | 6 weeks | Steroid pulse therapy | Ozaki K et al[4] |
| **7** | 53/M | NSCLC | Sintilimab | Haematuria,  pollakiuria and painful micturition | 7 weeks | Methylprednisolone | Lingfang Tu et al[5] |
| **8** | 60/M | Lung cancer | Nivolumab | Glans penile pain and micturition pain | 77 courses of nivolumab | Methylprednisolone and discontinuing nivolumab treatment | [Hiroyuki Fukunaga](https://pubmed.ncbi.nlm.nih.gov/?term=Fukunaga%20H%5BAuthor%5D) et al[6] |
| **9** | 48/M | ICC | Nivolumab | Urinary tract irritation | 3 cycles | Steroid hormones | Sihui Zhu et al[7] |
| **10** | 67/F | Advanced breast cancer | Atezolizumab | Urinary tract pain and frequent urination | 97 days | Prednisolone | [Aiko Obayashi](https://pubmed.ncbi.nlm.nih.gov/?term=Obayashi%20A%5BAuthor%5D) et al[8] |

NSCLC, non-small cell lung cancer; SCLC, small cell lung cancer; ICC, intrahepatic cholangiocarcinoma

**Reference**

**1** Zhu L, Wang Z, Stebbing J, Wang Z, Peng L: **Immunotherapy-Related Cystitis: Case Report and Review of the Literature**. *Onco Targets Ther* 2021, **14**:4321-4328.

**2** Ueki Y, Matsuki M, Kubo T, Morita R, Hirohashi Y, Sato S, Horibe R, Matsuo K, Tsukahara T, Kanaseki T *et al*: **Non-bacterial cystitis with increased expression of programmed death-ligand 1 in the urothelium: An unusual immune-related adverse event during treatment with pembrolizumab for lung adenocarcinoma**. *IJU Case Rep* 2020, **3**(6):266-269.

**3** Shimatani K, Yoshimoto T, Doi Y, Sonoda T, Yamamoto S, Kanematsu A: **Two cases of nonbacterial cystitis associated with nivolumab, the anti-programmed-death-receptor-1 inhibitor**. *Urol Case Rep* 2018, **17**:97-99.

**4**  Ozaki K, Takahashi H, Murakami Y, Kiyoku H, Kanayama H: **A case of cystitis after administration of nivolumab**. *Int Cancer Conf J* 2017, **6**(4):164-166.

**5** Tu L, Ye Y, Tang X, Liang Z, You Q, Zhou J, Pan Z: **Case Report: A Case of Sintilimab-Induced Cystitis/Ureteritis and Review of Sintilimab-Related Adverse Events**. *Front Oncol* 2021, **11**:757069.

**6**  Fukunaga H, Sumii K, Kawamura S, Okuno M, Taguchi I, Kawabata G: **A case of steroid-resistant cystitis as an immune-related adverse event during treatment with nivolumab for lung cancer, which was successfully treated with infliximab**. *IJU Case Rep* 2022, **5**(6):521-523.

**7** Zhu S, Bian L, Lv J, Liu B, Shen J: **A Case Report of Non-Bacterial Cystitis Caused by Immune Checkpoint Inhibitors**. *Front Immunol* 2021, **12**:788629.

**8** Obayashi A, Hamada-Nishimoto M, Fujimoto Y, Yoshimoto Y, Takahara S: **Non-bacterial Cystitis With Increased Expression of Programmed Cell Death Ligand 1 in the Urothelium: An Unusual Immune-Related Adverse Event After Atezolizumab Administration for Metastatic Breast Cancer**. *Cureus* 2022, **14**(5):e25486.
